# Partnerships in medical education: looking across disciplinary boundaries to extend knowledge

**DOI:** 10.1007/s40037-016-0261-9

**Published:** 2016-03-07

**Authors:** Emese Hall, Jennifer Cleland, Karen Mattick

**Affiliations:** Graduate School of Education, University of Exeter, Exeter, UK; Institute of Education for Medical and Dental Sciences, College of Life Sciences and Medicine, University of Aberdeen, Aberdeen, UK

In this issue of Perspectives on Medical Education, Sylvia Langlois [1] from the University of Toronto presents an initiative whereby health professions students worked with a health mentor, who was an individual experiencing chronic health challenges. Students reflected on their experiences of interacting with their mentor and one of the themes identified in the qualitative data was patient partnerships.

Langlois employed a range of phrases in reference to the partnerships promoted in her study, including: patient and family partnership; patient partnerships; patient-practitioner partnerships; patient-provider partnerships; personal relationship; team communication and collaboration. The term ‘partnership’ here could be seen to imply working together for a common purpose, on an even footing, as evident in the perspective of one of the students in Langlois’ study [1], who highlights the importance of recognizing ‘*the relationship in a partnership between the patient and the professional, and no one telling the other what to do and the other doing it without question*’. However, it could be argued that possible tensions in the partnership arrangement arise elsewhere in the paper, with the positioning of patients and practitioners as ‘clients’ and ‘providers’ respectively, giving rise to the question: to what extent should, or could, patient-practitioner partnerships be viewed as a truly reciprocal arrangement?

Moreover, reading this paper led us to think about the wide range of partnerships that exist within medical education, not only university/patient partnerships in delivering clinical skills teaching, but university/healthcare partnerships in delivering undergraduate programmes, and clinician/academic partners in undertaking research, to name but a few. We wondered whether there were existing definitions and categorizations that could help us to conceptualize the nature and range of partnerships in medical education. We were particularly interested in conceptualizing partnerships in a way which goes beyond serving the needs of one party, but which reflects equal reciprocity in terms of engagement and participation across parties.

We found that partnerships are typically defined by at least one of the partners having something that the other partner does not have. Ideally, a partnership would be mutually beneficial. For example, in the university/healthcare partnership in delivering undergraduate medical education, the university gains access to clinical expertise and placements in return for funding. Similarly, when clinicians undertake research together with an academic partner, they might provide clinical expertise and access to practitioners and patients in return for methodological and/or analytic expertise. There are clear reciprocal relationships here, of exchanging ‘things’ with others with benefits for each party. However, in other cases, what one of the partners stands to gain is less explicit. When patients are involved in delivering clinical skills teaching, the patient provides access to a body, possibly one with signs and symptoms, for students to examine and learn from—but what are the gains for them? Sometimes the gains are financial but often they are less tangible, as per the health mentors in Langlois’ study, who potentially benefitted a much wider group of future patients when students were sympathetic to their unsatisfactory healthcare experiences and keen to be mindful of these in their future clinical practice and advocacy. It seems important to think beyond the obvious rewards in partnerships in education and to articulate less tangible gains such as belief in the longer term impact of partnership.

Partnerships such as these are not unique to healthcare education and looking beyond the medical/healthcare education literature is important in considering different ways of thinking. For example, healthcare students working with patients is the equivalent of trainee teachers working with school pupils. Like medical education, partnerships in teacher training are a prerequisite and frequently exist between universities and schools. A review of recent international studies on partnerships in teacher education/training demonstrates wide-ranging interests in certain aspects of partnership. For example, some researchers have proposed models of partnership, with distinct ‘ingredients’ for success (e.g., Summers and Weir [2]). Others have investigated levels of engagement and partners’ perceptions (e.g., Bissaker [3]) and leadership as a component (e.g., Webster et al. [4]). In many cases partnership appears to be loosely understood as collaboration to facilitate authentic learning experiences with real-world outcomes. Only those researchers who propose or explain models of partnership actually offer some sort of definition of partnership. For example, in simple terms, Webster et al. [4] suggest that the partnerships in their study involved ‘*tapping into locally situated knowledge and harnessing the human resources of the university system and other community-based partners*’ (p. 197). We particularly like this framing of partnership as it moves thinking beyond the dominant partnership discourse in medical education, that of patient and public involvement or partnership (e.g., Regan de Bere and Nunn [5]).

A good start to encouraging critical exploration of partnerships is to agree a definition of what is meant by partnership in healthcare education, one which can be applied to partnerships in general, not just those involving patients. We would like to propose the following: partnership in medical education can be defined as: ‘*when two or more individuals/groups/organizations collaborate towards a shared goal of enhancing medical education, where one partner contributes something that the other(s) cannot provide*’. We further propose that equality and mutual respect between partners, and transparency around the nature of the partnership and responsibilities/benefits on each side, will be critically important elements of successful partnerships. We recognize that an even balance of power in the partners’ relationship, although desirable, is not always achievable (as indicated by the ‘client’ and ‘provider’ terms used by Langlois [1]); however, a partnership should have reciprocity at its heart, both as an ethical ideal and also a key driver for achieving positive outcomes, whether anticipated or unexpected.

Developing a robust pedagogic model of sustainable partnerships in medical education, by considering the conceptual, theoretical and empirical aspects of all partnerships relevant to professional education, would be a useful next step in advancing knowledge, practice and policy in this area.
